# A survey on adolescent health information seeking behavior related to high-risk behaviors in a selected educational district in Isfahan

**DOI:** 10.1371/journal.pone.0206647

**Published:** 2018-11-07

**Authors:** Somayeh Esmaeilzadeh, Hasan Ashrafi-rizi, Leila Shahrzadi, Firozeh Mostafavi

**Affiliations:** 1 Health Information Technology Research Center, Isfahan University of Medical Sciences, Isfahan, Iran; 2 Department of Health Education and Promotion, Faculty of Health, Isfahan University of Medical Sciences, Isfahan, Iran; Iran University of Medical Sciences, ISLAMIC REPUBLIC OF IRAN

## Abstract

**Backgrounds:**

The characteristics and conditions of growth and development have made adolescence one of the most vital and influential ages for prevention and health promotion, especially in the area of high-risk behaviors. Accordingly, the aim of this study was to determine adolescent health information seeking behavior related to high-risk behaviors in a selected educational district in Isfahan (Iran).

**Methodology:**

The present study was of an applied type, which was conducted using the survey research method. The statistical population consisted of adolescent students at public schools in Isfahan (6519 subjects), and the sample size was determined to be 364 based on Cochran's formula. The sampling method was of a cluster sampling type, and the data collection tool was a researcher-made questionnaire. The validity of the questionnaire was approved by medical librarians, and using the Cronbach's alpha method, the reliability was obtained to be 0.85. SPSS 16 software was used for data analysis at two statistical levels: descriptive and inferential (independent t-test, one-sample t-test, chi-square, Pearson correlation coefficient and Mann-Whitney).

**Findings:**

"Lack of mobility" was the most important health information need related to adolescent high-risk behaviors. The most important sources to obtain health information related to high-risk behaviors were "the Internet" with a mean score of 3.69 and "virtual social media" with a mean score of 3.49 out of 5. Adolescents had a positive attitude towards health information. The most important barriers to seeking health information were mentioned as follows: "difficulty in determining the quality of information found", "absence of appropriate information", and "concerns about the disclosure of their problems or illness to others". From the perspective of adolescents, the most important criterion for the evaluation of information quality was "the trueness and correctness of the information" and the need for health information related to high-risk behaviors was higher in girls than in boys.

**Conclusions/Significance:**

Considering adolescents’ positive attitude towards use of health information, it is necessary to put valid information at their disposal through different information resources, taking into account their level of information literacy. Accordingly, medical librarians’ abilities are suggested to be used for the production, evaluation, and introduction of health-related reading materials in the field of high-risk behaviors in easy language and suitable for adolescents.

## Introduction

Provision of health to adolescents is the basis of national development goals, and attention to adolescents and their exclusive health needs are among the goals of health services. This is a prerequisite for the health and development of mankind’s future, and any effort to promote health without consideration of adolescences will be incomplete [1]. On the other hand, statistics show that one-fifth of the world's population is between 10 and 19 years old, 85% of whom live in developing countries [2]. According to the latest report by the Statistical Center of Iran, and based on the 2016 Iranian census, adolescents were 12 million accounting for 8% of the country's population [3]. Adolescence is a period, during which environmental demands increase and a lot of serious mental disorders begin [4]. Changes occurring during this period of growth may cause certain problems, in a way that if the characteristics of people in this period are not properly directed, the ground will be prepared for the incidence of health threatening behaviors such as high-risk behaviors [5]. High-risk behaviors are defined as behaviors associated with some undesirable results [6]. Cigarette smoking, high-fat and low-fiber foods, physical inactivity, alcohol consumption, high-risk sexual behaviors, substance abuse, and injury and violence-related behaviors are regarded as high-risk behaviors [7–9]. In order to realize the goals of the Fifth Development Plan, they have compiled a document called the "Comprehensive Adolescent and Youth Health Plan" with an emphasis on social harms and high-risk behaviors, which, in turn, emphasizes the importance of addressing the issue of high-risk behaviors. Changing adolescents’ behavior require provision of information, informing the adolescents, and search for information [7]. Awareness of information about high-risk behaviors creates a group of needs in adolescents, some of which lead to information seeking behavior in the health domain. If a search for information is done for health purposes, it will be considered as a component of the decision-making process to adopt a health behavior, and will be called “health information seeking behavior” [10]. Health information seeking behavior refers to ways, through which people obtain information about health, diseases, health promotion, and health threatening behaviors, such as high-risk behaviors [11]. It is among medical librarians’ responsibilities to obtain more information about health information seeking in the area of high-risk behaviors, inform about different types of information sources, and validate high-risk behaviors. Because they play an essential role in delivering timely, valid, and accurate information to users, as well as evaluating the information and its sources. In fact, they are able to take effective steps to inform adolescents and prevent the incidence of these behaviors in them.

The importance of this issue is so great that research has been carried out both in the domain of health information seeking and in the domain of high-risk behaviors. Sultan, Joshua and Misra found out that printed and electronic sources were main factors for seeking health information. The Internet, family members, and physicians are common sources for health information, and WhatsApp and public websites are the most commonly used electronic sources for health information [12]. Yilma, Inthiran and Reidpath found out that students used the Internet and family members as the primary and secondary sources and physicians as the third source to obtain health information [13]. Stephens, Ryan and Cunningham found out that most adolescents spoke to a dentist or orthodontist to obtain dental information, speaking to peers and reading notices were other ways to obtain information, and few respondents had used the Internet [14]. Ybarra et al. found out that 81% of students used their parents, teachers, and other adults, 56% used books available in libraries, 50% used their siblings and friends, and 38% used the Internet and computers to find answers to their questions about health issues [15].

Merghati-Khoei et al. found out that addiction, high-risk sexual behaviors, and abnormal social behaviors (such as aggression) were risk behaviors mainly seen among high school boys. High risk sexual behaviors, early marriage, unhealthy eating habits, lack of mobility, and worries about the future (in terms of occupation, education, and marriage) were mainly seen in the girls' group. Although risk behavior priorities were different between boys and girls, but of these priorities, high-risk sexual behaviors were common between these two groups of students [16]. Esmaeilzadeh et al. studied the prevalence of high-risk behaviors among adolescents. The findings showed that hookah smoking had the highest prevalence among students in comparison with the experience of cigarette smoking and consumption of narcotics and alcohol. The prevalence of physical violence inside and outside the school during one year, carrying non-firearm weapons to school during one month, cigarette smoking at school during one month, and hookah smoking, drinking alcohol, and consumption of narcotics during one month were seen more in boys than in girls [17].

A review of the literature showed that no studies had been conducted on adolescent health information seeking behavior in Iran; also, there is no research on information seeking behavior associated with high risk behaviors abroad. Given that the adolescents' group are a suitable group for training their information tastes and guiding them towards correct health information seeking behavior related to adolescents’ high-risk behaviors, and considering the presence of a research gap in this regard, we conducted the present study with the aim of determining adolescent health information seeking behavior related to high-risk behaviors at selected public schools in Isfahan (Iran). Determining adolescent health information seeking behavior can seemingly help identify barriers to adolescents for accessing health information, identify health information resources used by adolescents, and provide managers and those involved in health information services with solutions to providing health services to adolescents.

## Methodology

The present study was of an applied type, which was conducted on Dec. 31, 2016 using the survey research method. The study population consisted of 6519 adolescent students at a selection of public schools in Isfahan. The schools are divided into two groups in Iran: public (state) and private schools. Since it was not possible to select both groups of schools due to the number and extent of the community, and the number of public schools was more than the private ones, and public schools were less-equipped, the doctors decided to select only the public schools because this selection would help homogeneity of society and generalization of the results. Moreover, all the teenager students aged between 15 and 18 of the public schools in District 2 in Isfahan, who were studying in September 2017 in the above-said schools, and who wanted to participate in the research were considered as the inclusion criteria, and the dissatisfaction of students and parents and failure to complete the questionnaire were considered as the exclusion criteria.

We determined the sample size to be 364 using Cochran’s formula. The sampling method was of a cluster sampling type. In fact, Isfahan has six educational districts, out of which we randomly selected one district (District 2). In fact, we used cluster sampling to select one district out of 6 because it was not possible to study all the districts due to high number of districts as well as wide geographical distribution. Indeed, cluster sampling is the best sampling method in these situations; hence, only one district was selected randomly (District 2). In the next stage, we selected boy and girl students in the second grade at six high schools in District 2, and administered the questionnaires among them in person. The data collection tool was a researcher-made questionnaire based on questionnaires developed by Keshavarz, Shabani, and Vasfi [18]; Zare Gavghani, Ghaysari, and Asghari Jafar-Abadi (10); Bigdeli, Hayati, Jokar, and Heidari [19], and Dastani and Mohammadi [20] (S1 Text). Since no questionnaire has been found on this topic, the researchers conducted an extensive search in the information databases in Persian and English languages. Finally, few relevant articles were found. The articles were studied by the researchers and the introductory questionnaire was prepared. In fact, this questionnaire was examined by several other specialists and the students in addition to the research team. In the end, the research team prepared the introductory questionnaire based on their comments. In order to evaluate the reliability of the tools, 10% of the sample size including 40 students was selected and the tools were completed by them. The data were entered in the SPSS software and the tools' reliability was obtained to be 0.85. The process of preparation of questionnaire started from April 3, 2017 and ended on June 29, 2017. To make sure that the questionnaires would be completed, we distributed a sufficient number of questionnaires (400 questionnaires) among students, out of whom 392 students completed the questionnaires. The process of completion of questionnaire started from September 23, 2017 and ended on December 10, 2017. Before that, the required correspondence was carried out with the Education Department of Isfahan Province, and the necessary licenses were obtained. Moreover, all transportation costs for the completion of the questionnaires by the researchers were calculated and paid based on their destination, number of trips during the research, and the type of vehicle used. This questionnaire consisted of the following items: demographic information (Items 1 to 7), questions about the identification of needs and the history of medical and health information seeking related to high-risk behaviors (Items 8 to 11), questions about medical and health information seeking resources related to high-risk behaviors (Items 12 to 17), a question about the attitude of the study population towards the importance of medical and health information related to high-risk behaviors (Item 18), a question about barriers to access medical and health information related to high-risk behaviors (Item 19), and a question about the validation of medical and health information related to high-risk behaviors (Item 20). With regard to the nature of research, in some questionnaires, yes/no questions were used, and in most cases, 5-choice Linkert spectrum was used. The mean and higher ranks indicated higher importance of that particular element as far as the research population was concerned. The validity of the questionnaire was approved by medical librarians. The medical librarians were selected as mediators between health information sources and users. Moreover, they conduct most of researches on the parameters of health information behavior and effective variables. Therefore, these individuals are qualified to determine the validity of health information behavior evaluation tools, and the existing researches prove this. Using the Cronbach's alpha method, the reliability of the questionnaire was calculated to be 0.85 (S1 Table). Data were collected through in-person visits, and ethical principles were taken into consideration during the research. This research was conducted in accordance with the Declaration of Helsinki. All research procedures and protocols including participant recruitment materials were reviewed and approved by Ethics Committee on Medical Research, Deputy of Research and Technology at Isfahan University of Medical Sciences, Isfahan, Iran. The members include 10 health professionals, such as medical education, Pediatric, cardiology, digestion, midwifery, Islamic education and medical ethics. Parents of participating subjects provided consent and all participating adolescents provided verbal assent. In Iran, to investigate the students under 18, parents' consent is needed. Therefore, in addition to obtaining the necessary licenses from the relevant organization, written and oral consents were obtained from the parents and students, respectively. We used the data analysis software SPSS 16 for data analysis at two statistical levels: descriptive (frequency, frequency percentages, mean, and standard deviation) and inferential (independent t-test, one-sample t-test, chi-square, Pearson correlation coefficient, and Mann-Whitney). In this research, Mann-Whitney parametric and non-parametric tests were used proportionate to the research questions. Non-parametric test was used to compare the level of the curiosity and tendency to search for health information related to high-risk behaviors among girls and boys as this test seems to be appropriate to identify the difference in scores of one or more variables between two independent groups whose variables are qualitatively ordinal. In other words, it is the most general test to determine the difference between distributions of two independent samples. Single-sample t-test was used to obtain the mean score of the students' attitude towards the importance of medical health information related to high-risk behaviors because single-sample t-test is a parametric test, which is used when we want to compare the mean of a sample of community with a normal and standard state, or even an expected number (in this research, the average limit is determined to be 50). In addition Pearson correlation coefficient was used to show the relation between the curiosity and tendency to search for health information related to high-risk behaviors, the attitudes towards the importance of health information related to high-risk behaviors, and barriers to obtaining health information related to high-risk behaviors, and age and level of income of adolescent’ families because Pearson correlation coefficient is used to determine the relation between two variables and their interaction, which is between -1 and +1, where zero shows the absence of relation between them. Chi-square test is also used to determine the relation between adolescent's' sex and their health information behavior (S2 Table).

## Findings

Interestingly, the return rate for the questionnaire was 100%. In terms of gender, girls made up 51.8% of the population, and boys 48.2%. The studied adolescents were ranging in age from 15 to 18 years, and the mean value of their families’ monthly income was Rls. 26,445,600 ($ 755). From among the adolescents, 73.7% were familiar with a language other than Persian, and 75.5% had needed medical and health information during the past six months.

The findings regarding medical and health information seeking related to adolescent’s high-risk behaviors for different purposes showed that the highest mean scores (out of 5) belonged to the options; information seeking for the "adolescent themselves" with a mean score of 2.95 followed by information seeking for the "parents" with a mean score of 2.36, and the lowest mean score belonged to information seeking for "classmates" which was 1.36.

The findings regarding health information needs showed that pieces of information about "lack of mobility" (3.14 for boys and 3.36 for girls), "high-risk sexual behaviors" (2.62 for boys and 2.34 for girls), and "incidents and injuries" (2.59 for boys and 2.27 for girls) were the most important health information needs (the scores were calculated out of 5). And the Mann-Whitney test showed that the curiosity and willingness to seek health information on the subjects; incidents and injuries (p = 0.04) and physical violence (p = 0.006) were significantly higher in boys than in girls, but in the other subjects, no significant differences were observed between girls and boys.

The most important sources to obtain health information related to high-risk behaviors performed by the studied adolescents are "the Internet" (3.69) and "virtual social media" (3.49), and the least important source to obtain health information related to high-risk behaviors is "radio” with a mean score of 1.43 out of 5 (Table 1).

**Table 1:** The ranks and mean scores of health information sources related to adolescents’ high-risk behaviors.

The health information sources related to adolescents’ high-risk behaviors	Mean score (out of 5)	Rank
The physician or other members of the treatment staff	2.50	4
TV	2.47	5
Radio	1.43	12
Friends or classmates	2.23	7
The Internet	3.69	1
Virtual social media	3.49	2
The teachers and school officials	1.90	9
Mobile applications	2.43	6
Information sources available in public libraries	1.88	10
Family members (father, mother, sisters, brothers, etc.)	3	3
Satellite channels	2.09	8
Workshops and meetings on health	1.58	11

The research findings regarding the criteria for the selection of health information sources showed that "accessibility", "higher reliability in terms of information", and "confidentiality of information" (with means of 42.2, 36.4 and 15.3 out of 100, respectively) were the most important criteria for the selection of health information sources related to high-risk behaviors.

The findings regarding the place to start the search for health information showed that from among the 293 adolescents who had completed this part of the questionnaire, 232 (59.2%) used the Internet to obtain health information related to high-risk behaviors during the past month, out of whom 52.8% chose "search engines" and 16.3% chose "social media" as places where they usually started their search on the Internet, and 47.7% of the adolescents who had not searched for health information on the Internet during the past month, referred to the “unreliability of the Internet" as their reason for not using the Internet.

The findings showed that the most difficult points and barriers to adolescents for accessing and using health information related to high-risk behaviors were "difficulty in determining the quality of information found" (2.73) and "absence of appropriate information" (2.68), and the least important difficulty and barrier was "being punished by their parents or school officials” (1.82) (Table 2).

**Table 2:** Ranks and mean scores of difficulties and barriers to adolescents for accessing health information related to high-risk behaviors.

Difficulties and barriers	Mean score (out of 5)	Rank
Lack of access to appropriate and practical information sources in a simple language	2.44	5
Concerns about the disclosure of their problems or illness to others	2.49	3
High costs of access to medical and health information	2.47	4
Believing that they can solve the problem or the disease themselves.	2.37	6
Being punished by their parents or school officials	1.82	8
Lack of information or inability to find the information being searched for	2.33	7
Difficulty in determining the quality of information found	2.73	1
The absence of proper information	2.68	2

The findings showed that the most important criteria for the quality of health information related to high-risk behaviors were "the trueness and correctness of the information" (4.02) and "validity and reliability of the information” (3.97), and the least important criterion was "the availability of the author's postal address and phone number” (2.29) (Table 3).

**Table 3:** Mean scores of the criteria for the quality of health information related to high-risk behaviors from adolescents’ perspective.

The criteria for the quality of information	Mean score (out of 5)	Rank
The expertise, experience, and reputation of the author of the content	3.18	10
The availability of the author's postal address and phone number	2.29	16
The author's dependence on a reputable and prestigious institute	2.65	15
The simplicity of finding the information	3.47	6
Free access to information	3.38	8
Provision of information about the terms and conditions of accessing and using the content	2.69	14
Providing the date of publishing the content	2.88	12
Keeping the information up-to-date	3.75	4
Impartiality and absence of bias	2.94	11
The trueness and correctness of the information	4.02	1
Validity and reliability of the information	3.97	2
The breadth and scope of the information	3.45	7
Understandability of the information content	3.92	3
Provision of new and innovative information	3.70	5
Taking the audience into consideration	3.23	9
A friend's recommendation to use a type of information	2.84	13

The findings on the mean scores of the curiosity and tendency to search for health information related to high-risk behaviors, the attitudes towards the importance of health information on high-risk behaviors, and barriers to obtaining health information related to high-risk behaviors (out of 100), by gender variable using the independent t-test, showed that there were no significant differences between girls and boys in the mean scores of the curiosity and tendency to search for health information on high-risk behaviors (p = 0.20), attitudes towards the importance of health information related to high-risk behaviors (p = 0.06), and barriers to obtaining health information related to high-risk behaviors (p = 0.73). In other words, the mean scores of the curiosity and tendency to search for health information related to high-risk behaviors, the attitudes towards the importance of health information related to high-risk behaviors and barriers to obtaining health information related to high-risk behaviors between boys and girls is same.

And the Pearson correlation coefficient between the mean scores of the curiosity and tendency to search for health information related to high-risk behaviors, the attitudes towards the importance of health information related to high-risk behaviors, and barriers to obtaining health information related to high-risk behaviors on the one hand, and the age and income levels of the adolescents’ families on the other hand, showed that there was no significant correlation between the mean scores of the curiosity and tendency to search for health information related to high-risk behaviors (p = 0/43), the attitudes towards the importance of health information related to high-risk behaviors (p = 0/37), and barriers to obtaining health information related to high-risk behaviors(p = 0/31) and the age and income levels of the adolescents’ families. In other words, the mean scores of the curiosity and tendency to search for health information related to high-risk behaviors, the attitudes towards the importance of health information related to high-risk behaviors, and barriers to obtaining health information related to high-risk behaviors between different age groups and also different income levels of the adolescents’ families is same.

Analyzing the data through independent t-test showed that there were no significant differences between “adolescents familiar with a non-Persian language” and “adolescents unfamiliar with a non-Persian language” in the mean scores of the curiosity and tendency to search for health information related to high-risk behaviors (p = 0.49), the attitudes towards the importance of health information related to high-risk behaviors (p = 0.73), and barriers to obtaining health information related to high-risk behaviors (p = 0.94).

The findings regarding the need for health information related to high-risk behaviors in adolescents, by their gender using the chi-square test, showed that the need for health information related to high-risk behaviors in the last six months was significantly higher in girls than in boys (p- value < 0.001).

## Discussion

The findings showed that the most important needs for health information related to high-risk behaviors among adolescents were the needs for obtaining information about "lack of mobility", "high-risk sexual behaviors", and "incidents and injuries". This finding was consistent with the results of a study by Merghati-Khoei et al. on the prioritization of high-risk health behaviors among students [16], a study by Obasola and Agunbiade on the online health information seeking behavior pattern among undergraduate students [21], and a study by Garmaroudi et al. on high-risk health habits among students [22], and was inconsistent with the findings of a study by Esmaeilzadeh et al. on the prevalence of high-risk behaviors among adolescents [17]. The most important reason for the inconsistency of the findings can be the difference between the characteristics of the statistical populations and the places where the studies carried out. Only the degrees of needing information on "incidents and injuries" and on “physical violence" were higher in boys than in girls, and no differences were observed between boys and girls in the other areas.

The findings showed that respondents referred to the "Internet", "social media", and "the family" as the most important sources for obtaining health information related to high-risk behaviors. This finding was consistent with the results of a study by Okhovati et al. on health information seeking behavior and the role of public libraries [23], a study by Bigdeli et al. on the place of the Internet in health information seeking behavior [19], a study by Sultan, Joshua and Misra on health information seeking behavior among students [12], a study by Gray et al. on adolescent health information seeking behavior on the Internet [24], a study by Michele et al. on health information seeking among adolescents [15], a study by Renahy, Parizot, and Chauvin on health information seeking on the Internet [25], a study by George Ettel et al. on how adolescents should access health information [26], a study by Owsusu-Addo, Owsusu-Addo and Morhe on health information seeking behaviors among pregnant adolescents [27], a study by Simou on health information sources [28], a study by Nolke et al. on the sociological characteristics of online health information seeker [29], a study by Yilma, Inthiran and Reidpath on places where university students obtain health information in developing countries [13], a study by Shamsi et al. on health information seeking behavior among elementary school teachers [30], and was inconsistent with a study by Noh et al. on cervical cancer patients’ information-seeking behaviors, information needs, and information sources [31], a study by Stephens, Ryan and Cunningham on health information seeking behavior, health indicators, and health risks [14]. The different types of statistical populations, which generally consisted of patients, and the different places where the studies were conducted, could be the most important reasons for this inconsistency. The results also showed that the place to start the search for health information related to high-risk behaviors on the Internet was search engines, which was consistent with the results of a study by Zare Ghavghani, Ghaysari, and Asghari Jafar Abadi on health information seeking behavior among the members of public libraries in Qazvin [10], a study by Sultan, Joshua and Misra on health information seeking behavior [12], and a study by Yilma, Inthiran and Reidpath on places where university students obtain health information in developing countries [13].

The findings showed that adolescents had positive attitudes towards the importance of health information related to high-risk behaviors. This result was consistent with the results of a study by Zamani et al. on cardiovascular patients’ information seeking behavior [32], a study by Zare Gavghani, Ghaysari, and Asghari Jafar Abadi on health information seeking behavior among the members of public libraries [10], a study by Bigdeli et al. on the place of the Internet in health information seeking behavior [19], and a study by Weaver et al. on health information seeking behavior, health indicators, and health risks [33].

The findings showed that "the difficulty in determining the quality of information", "absence of appropriate information", and "concerns about the disclosure of problems or illness to others" were respectively the most important problems for adolescents to seek for health information. This finding was consistent with the results of a study by Keshavarz, Shabani, and Vasfi on the validation of health information available on the Web [18], and a study by Nasrollahzadeh on pregnant women’s health information seeking behavior [34], and was inconsistent with the result of a study by Numark, Allen and Starkshall on adolescent’s online health information seeking [35]. The most important reasons for the inconsistency can be the difference between barriers to accessing online health information sources and barriers to accessing other health information sources, as well as the different places where the studies were carried out.

Based on the research findings, the most important criteria for the quality of health information related to high-risk behaviors, from adolescents’ perspective, were "the trueness and correctness of the information", "validity and reliability of the information", and "understandability of the information content", respectively. This finding was consistent with the results of a study by Nasrollahzadeh on pregnant women’s health information seeking behavior [34], and was inconsistent with the results of a study by Keshavarz, Shabani, and Vasfi on the validation of health information available on the Web [18]. The most important reason for the inconsistency can be the difference in the criteria for the quality of information in printed and online sources.

The findings showed that there was a significant relationship between gender and the need for health information related to high-risk behaviors in adolescents, and that girls are more in need of health information. This finding was consistent with the results of a study by Bigdeli et al. on the place of the Internet in health information seeking behavior [19], a study by Okhovati et al. on health information seeking behavior and the role of public libraries [23], a study by Lee et al. on adolescent health information seeking behavior on the Internet [36], and a study by Otowmbe et al. on sexual health information seeking behavior among adolescents [37].

Based on the findings, there was no significant relationship between gender and the mean scores of the attitudes towards the importance of health information related to high-risk behaviors, the curiosity and tendency to search for health information related to high-risk behaviors, and barriers to seeking health information related to high-risk behaviors among adolescents. This finding was consistent with the results of a study by Zamani et al. on cardiovascular patients’ information seeking behavior [32], a study by Rahimianfar, Hakimian, and Salimi on information seeking behavior among nurses [38], a study by Sultan, Joshua and Misra on health information seeking behavior among students [12], and was inconsistent with the results of a study by Bigdeli et al. on the place of the Internet in health information seeking behavior [19], a study by Okhovati et al. on health information seeking behavior and the role of public libraries in Kerman [23], a study by Numark, Allen and Starkshall on adolescents’ online health information seeking [35], a study by Lee et al. on adolescents’ online health information seeking behavior [36], a study by Otowmbe et al. on sexual health information seeking behavior among adolescents [37], a study by Nolke et al. on the sociological characteristics of online health information seekers [29]. The reason for the inconsistency of the findings seems to be that most studies in this area have merely been investigated health information seeking behavior in online sources.

Based on the findings, there was no significant relationship between age and the mean scores of the attitudes towards the importance of health information related to high-risk behaviors, the curiosity and tendency to search for health information related to high-risk behaviors, and barriers to seeking health information related to high-risk behaviors among adolescents. This finding was consistent with the results of a study by Sultan, Riju and Misra on health information seeking behavior among students [12], and was inconsistent with the results of a study by Lalehzarian et al. on the effects of individual factors on diabetic patients’ health information seeking behavior [11], a study by Okhovati et al. on health information seeking behavior and the role of public libraries in Kerman [23], and a study by Lee et al. on online health information seeking behavior [36]. The slight difference in age between the studied adolescents can be a reason for the inconsistency.

Based on the findings, there was no significant relationship between the income levels and the mean scores of the attitudes towards the importance of health information related to high-risk behaviors, the curiosity and tendency to search for health information related to high-risk behaviors, and barriers to seeking health information related to high-risk behaviors among adolescents. This result was inconsistent with the results of a study by Renahy, Parizot, and Chauvin on health information seeking on the Internet [25], and a study by Nolke et al. on the sociological characteristics of online health information seekers [29].

One of the limitations of this study was the absence of resources on adolescent health information seeking behavior in Iran, especially in the field of high-risk behaviors. To overcome this obstacle, we used resources about adolescent health information seeking behavior in foreign sources, as well as sources which were slightly similar in methodology or subject. The strength of this study is its statistical population; that is adolescents. Because adolescents and students can have a tremendous impact on the health of the family and society, they are usually able to transfer health messages and habits from school to home, and promoting their awareness and improving their attitude can lead to positive changes in people around them. Since just one district has been selected from among the six educational districts, the results must be generalized with a higher level of precision.

## Conclusion

It is important to pay attention to social behaviors and lifestyle of adolescents due to their impact on the quality of their life; and health knowledge is the background to health behaviors. Therefore, a healthy lifestyle due to increased health knowledge can improve the quality of life of individuals and cause minimum illness, disability, and injury. The important point here is that health behaviors such as high-risk behaviors are different in adolescence compared to adulthood, so knowing the mental structure and attitude of adolescents and trying to use health and medical information allow assessing the lifestyle in a precise manner based on their main essence, and providing them with valid health information through different information sources regarding the level of skill and informational literacy. Moreover, increasing the adolescents’ level of knowledge, new and proper preventive approaches can be developed, adolescents’ abilities can be promoted, and their unhealthy behaviors can be modified. According to the results obtained in this research, in general, adolescents have a positive attitude towards health information, and believe that it is useful. Inactivity is the most important health information requirement related to high-risk behaviors of adolescents. This information may be acquired through different channels such as medical teams, official or unofficial channels, traditional channels, and media channels. According to the findings of this research, difficulty in determining the quality of information found, lack of suitable information, and concern about the disclosure of the problem or disease to others are the most important problems in the search for medical and health information related to high-risk behaviors among teenager students. The internet and social media are the most important information sources used by adolescents to obtain health information related to high-risk behaviors. According to the features of the society under study, development of modern technologies, increased access to the internet, and development of the social media in recent years, this finding is natural to a large extent. The most important criteria to evaluate the quality of information in the view of adolescents include the accuracy of information, its validity and understandability. According to the findings, the need for health information related to high-risk behaviors in girls was more than that in boys. This is while there was no significant relationship between the mean scores of the curiosity and tendency to search for health information related to high-risk behaviors, the attitudes towards the importance of health information related to high-risk behaviors, and barriers to obtaining health information related to high-risk behaviors and sex, age, household income level, and understanding non-Persian languages. Regarding the positive attitude of adolescents towards using health information, it is necessary to provide them with valid information through different information sources in accordance with the adolescents’ information literacy level. This can have a significant role in the preventive activities and reduction of medical costs. It can also minimize the disability, injury and illness. In order to obtain health information, adolescents stated barriers such as difficulty in the determination of the quality of information and lack of suitable health information indicating the need for effective measures in this field. To overcome these obstacles and problems, it is necessary to design databases, websites, and valid apps through official centers and institutes by the Ministry of Health and Medical Education, and Ministry of Education. On the other hand, through providing trainings about the health and media literacy, creating native health databases proportionate to the age group of adolescents, and identifying and introducing valid resources in the field of health information, especially adolescents’ high-risk behaviors, we can try to overcome these obstacles. Accordingly, the following solutions are suggested:

- Since adolescents use the internet and social media to gain health information related to high-risk behaviors, the practitioners, especially medical librarians and information providers, are obliged to try more to create valid information contexts in these fields and provide simple health reading materials for these environments.
- Due to the plurality and diversity of information sources used by the adolescents to obtain health information related to high-risk behaviors on the one hand, and sensitivity of this kind of information on the other hand, it is necessary for the adolescents to have enough media literacy to use different media more effectively in order to obtain health information. Therefore, holding training course, providing training brochures related to media literacy and high-risk behaviors are suggested.
- According to the findings of the research, one of the most important barriers to gain health information by the adolescents is the difficulty in determining the quality of information found. Therefore, it is recommended to consider trainings for the methods and strategies for health information search, evaluation of the quality of information found, and introduction of the health information sources to adolescents through libraries and valid training centers.
- Utilizing the abilities and capabilities of medical librarians and information providers in order to train health and media literacy can be an important factor in acquiring update and useful health information for adolescents.
- Evaluating useful and valid websites in the area of health by health information specialists, especially medical librarians and information providers and introducing them to adolescents is an important factor in the secure access to valid health information.
- Providing necessary trainings about the high-risk behaviors through libraries, especially training and public libraries, and proportionate training sources along with these trainings.
- Considering high-risk behaviors and how to prevent them in the training programs.

## Supporting information

S1 Text Questionnaire in English. (DOCX)

S1 Table Cronbach's alpha. (PDF)

S2 Table Raw data. (SAV)
