# Supplementary material for: A survey on adolescent health information seeking behavior related to high-risk behaviors in a selected educational district in Isfahan
Source: PLoS One. 2018 Nov 7;13(11):e0206647. doi: 10.1371/journal.pone.0206647 (PMC6221342; doi:10.1371/journal.pone.0206647)
Supplement: S1 Text — (DOCX) [file pone.0206647.s001.docx]

**S 1 questionnaire**

Dear student:

Hello,

We are pleased to present you with a questionnaire considered for conducting a study titled “A survey on adolescent health information seeking behavior related to high-risk behaviors in a selected educational district in Isfahan”. Please help us better carry out this study by answering the questions carefully. Meanwhile, we want to point out that the information received is for the sole purpose of this study, and that this information will be kept confidential by the researcher. We really appreciate your kindness and cooperation.

An explanation of some of the concepts:

- Health information: Any kind of medical and health information that helps you in preventing, treating, and recovering from an illness, and that warns you about behaviors such as: consumption of narcotics, inappropriate nutrition, violence, lack of mobility, incidents and injuries, and high-risk sexual behaviors
- Health information sources: Wherever an individual can obtain the information he or she needs, such as friends, classmates, the family, physician, printed sources, such as books, magazines, brochures, newspapers, etc., and non-printed sources, such as television, radio, Internet websites, etc.
- Health information seeking: Any kind of activity that helps you obtain medical and health information, such as visiting a physician, asking questions of others, watching TV, listening to radio, searching the Internet, visiting a library, etc.

**a) Demographic Characteristics**

1. **Gender:** Girl 🌕 Boy 🌕
2. **Age:**
3. **Level of education:** ………
4. **Address or neighborhood name:** ..............................
5. **The average monthly income of your family:** ................. (in Iranian tomans)
6. **Are you familiar with a language other than Persian (English, German, Arabic, etc.)? Yes 🌕 Name them:** ................... No 🌕
7. **What is your degree of competence in the language you mentioned in the previous question?**

Very high 🌕 High 🌕 Moderate 🌕 Low 🌕 Very low 🌕

**b) History of medical and health information seeking**

1. **Have you needed medical and health information during the last six months.**

Yes 🌕 No 🌕

(If your answer is no, only answer question 9, if yes, go to question 10 and the next questions.)

1. **If your answer to question 8 was “No”, choose one of the following options:**

a) I have not felt any need for medical and health information. 🌕

b) I have felt the need for medical and health information, but I have not had time to seek for the information. 🌕

c) I have felt the need for medical and health information, but I was not familiar with health information sources. 🌕

d) I have felt the need for medical and health information, but I have not had access to health information sources. 🌕

Other reasons: ...........................

1. **Whom you have more sought medical and health information for in the last six months?**

|  | **Very slightly** | **Slightly** | **Moderately** | **Greatly** | **Very greatly** |
| --- | --- | --- | --- | --- | --- |
| **For myself** |  |  |  |  |  |
| **For my parents** |  |  |  |  |  |
| **For my siblings** |  |  |  |  |  |
| **For relatives and acquaintances** |  |  |  |  |  |
| **For other members of my family** |  |  |  |  |  |
| **For my friends** |  |  |  |  |  |
| **For my classmates** |  |  |  |  |  |
| **Other (*Please name them*):** | **.......................................** | | | | |

1. **Which of the following health and medical subjects are your curious about and willing to seek for? And to what extent?**

|  | **Very slightly** | **Slightly** | **Moderately** | **Greatly** | **Very greatly** |
| --- | --- | --- | --- | --- | --- |
| **Incidents and injuries (accidents, falls from height, injuries, etc.)** |  |  |  |  |  |
| **Tobacco abuse (cigarettes, hookahs, etc.)** |  |  |  |  |  |
| **Lack of physical activity (sports, fitness, etc.)** |  |  |  |  |  |
| **Narcotics and alcohol (alcohol or drug rehabilitation)** |  |  |  |  |  |
| **Violence (physical violence)** |  |  |  |  |  |
| **High-risk sexual behaviors (HIV, sexual diseases, etc.)** |  |  |  |  |  |
| **Other (*Please name them*):** | **.........................................** | | | | |

**c) Sources for seeking medical and health information**

1. **How do you obtain medical and health information related to high-risk behaviors?**

|  | **Very slightly** | **Slightly** | **Moderately** | **Greatly** | **Very greatly** |
| --- | --- | --- | --- | --- | --- |
| **Visiting the physician or other members of the treatment staff** |  |  |  |  |  |
| **Watching TV (medical series, medical news, interviews with physicians)** |  |  |  |  |  |
| **Listening to radio** |  |  |  |  |  |
| **Asking questions of friends or classmates** |  |  |  |  |  |
| **Searching the Internet** |  |  |  |  |  |
| **Using virtual social media such as: Telegram, Instagram, Facebook, Twitter, etc.** |  |  |  |  |  |
| **Asking questions of teachers and school officials** |  |  |  |  |  |
| **Using applications installed on your cellphone or tablet** |  |  |  |  |  |
| **Using information sources available in public libraries** |  |  |  |  |  |
| **Asking questions of your family members (father, mother, sisters, brothers, etc.)** |  |  |  |  |  |
| **Watching satellite channels** |  |  |  |  |  |
| **Attending workshops and meetings on health** |  |  |  |  |  |
| **Other (*Please name them*):** | **....................................................** | | | | |

1. **Which of the following reasons has had the greatest effect on your choice in the previous question?** (Choose only one option.)

Ease of access 🌕 Higher reliability 🌕 Lower costs 🌕 Confidentiality of information 🌕

Other (*Please name them*): .................................

1. **Have you used the Internet to seek for medical and health information in the last month?**

**Yes** 🌕 No 🌕

1. **If your answer to the above question was negative, what has been your reason for not using the Internet for medical and health information?**

Unreliability 🌕 Lack of access to the Internet 🌕 Incompetence to search for and use Internet information

Other (*Please name them*):

1. **In case you obtain medical and health information from the Internet, which of the following is usually the place you start your search? (Choose only one option.)**

🌕 A search engine such as Yahoo, Google, etc. (name an example you usually use): ...........................................

🌕 A specific website in the health domain (name an example you usually use): .............................................
🌕 The website of a healthcare center (name an example you usually use): ...............................

🌕 Social media such as: Cloob, Facebook, Telegram, WhatsApp, Instagram, etc.(name an example you usually use):**..............**

🌕 Weblogs (name an example you usually use): .......................

🌕 Electronic discussion groups (name an example you usually use): ........................
🌕 Other (*Please name them*): ...........................................

1. **Do you search for the health information you need in languages other than Persian too (e.g., in English)?**

🌕 Always 🌕 Often 🌕 Sometimes 🌕 Rarely 🌕 Never

**d) Your attitude towards medical and health information**

1. **Taking into account your experiences and attitude towards medical and health information related to high-risk behaviors, to what extent do you agree with the following statements?**

- Useful and valid medical and health information can be obtained easily.

Strongly agree 🌕 Agree 🌕 Agree to some extent 🌕 Disagree 🌕 Strongly disagree 🌕

- Seeking medical and health information helps me understand the conditions, my health status, and possible treatments required.

Strongly agree 🌕 Agree 🌕 Agree to some extent 🌕 Disagree 🌕 Strongly disagree 🌕

- Seeking medical and health information reduces my anxiety and worries about my and my acquaintances’ health.

Strongly agree 🌕 Agree 🌕 Agree to some extent 🌕 Disagree 🌕 Strongly disagree 🌕

- Seeking medical and health information gives me more confidence to manage my and my acquaintances’ health.

Strongly agree 🌕 Agree 🌕 Agree to some extent 🌕 Disagree 🌕 Strongly disagree 🌕

- Seeking medical and health information gives me more confidence to communicate with the physician and the treatment staff.

Strongly agree 🌕 Agree 🌕 Agree to some extent 🌕 Disagree 🌕 Strongly disagree 🌕

**e) Barriers to accessing medical and health information**

1. **What are your problems and obstacles when seeking medical and health information related to high-risk behaviors?**

|  | **Low** | **Very low** | **Moderate** | **High** | **Very high** |
| --- | --- | --- | --- | --- | --- |
| **Lack of access to appropriate and practical information sources in a simple language** |  |  |  |  |  |
| **Concerns about the disclosure of their problems or illness to others** |  |  |  |  |  |
| **High costs of access to medical and health information** |  |  |  |  |  |
| **Believing that they can solve the problem or the disease themselves.** |  |  |  |  |  |
| **Being punished by their parents or school officials** |  |  |  |  |  |
| **Lack of information or inability to find the information being searched for** |  |  |  |  |  |
| **Difficulty in determining the quality of information found** |  |  |  |  |  |
| **The absence of proper information** |  |  |  |  |  |
| **Other (*Please name them*):** | **....................................................** | | | | |

**f) The validity of medical and health information**

1. **When evaluating medical and health information related to high-risk behaviors, how important is each of the following items?**

| **The criteria for the quality of information** | | **Never** | **Rarely** | **Sometimes** | **Often** | **Always** |
| --- | --- | --- | --- | --- | --- | --- |
| **1** | **The expertise, experience, and reputation of the author of the content** |  |  |  |  |  |
| **2** | **The availability of the author’s phone number and postal address** |  |  |  |  |  |
| **3** | **The author's dependence on a reputable and prestigious institute** |  |  |  |  |  |
| **4** | **The simplicity of finding the information** |  |  |  |  |  |
| **5** | **Free access to information** |  |  |  |  |  |
| **6** | **Provision of information about the terms and conditions of accessing and using the content (the observance of copyright)** |  |  |  |  |  |
| **7** | **Providing the date of publishing the content** |  |  |  |  |  |
| **8** | **Keeping the information up-to-date** |  |  |  |  |  |
| **9** | **Impartiality and absence of bias (favoritism or supporting a certain person or organization more than others)** |  |  |  |  |  |
| **10** | **The trueness and correctness of the information** |  |  |  |  |  |
| **11** | **Validity and reliability of the information** |  |  |  |  |  |
| **12** | **The breadth and scope of the information (the superficial or profound presentation of the content)** |  |  |  |  |  |
| **13** | **Understandability of the information content** |  |  |  |  |  |
| **14** | **Provision of new and innovative information** |  |  |  |  |  |
| **15** | **Taking the audience into consideration (proportionally to age groups such as: school students, adults, etc.)** |  |  |  |  |  |
| **16** | **A friend's recommendation to use a type of information; e.g., watching a certain satellite channel or joining a Telegram group** |  |  |  |  |  |
