# Supplementary material for: A survey on adolescent health information seeking behavior related to high-risk behaviors in a selected educational district in Isfahan
Source: PLoS One. 2018 Nov 7;13(11):e0206647. doi: 10.1371/journal.pone.0206647 (PMC6221342; doi:10.1371/journal.pone.0206647)
Supplement: S1 Table — (PDF) [file pone.0206647.s002.pdf]

```
 GET
  FILE='C:\Users\Ali\Desktop\Esmaeilzade_S9608\data.sav'.
DATASET NAME DataSet1 WINDOW=FRONT.
RELIABILITY
  /VARIABLES=X10_1 X10_2 X10_3 X10_4 X10_5 X10_6 X10_7 X11_1 X11_2 X11_3 X11_4 X11_5 X11_6 X12_
X20_3 X20_4 X20_5 X20_6 X20_7 X20_8 X20_9 X20_10 X20_11 X20_12 X20_13 X20_14 X20_15 X20_16
  /SCALE('ALL VARIABLES') ALL
  /MODEL=ALPHA.
```

Reliability

[DataSet1] C:\Users\Ali\Desktop\Esmaeilzade_S9608\data.sav

Scale: ALL VARIABLES

Case Processing Summary

		N	%
Cases	Valid	168	42.9
	Excluded[a]	224	57.1
	Total	392	100.0

a. Listwise deletion based on all variables in the procedure.

Reliability Statistics

Cronbach's Alpha	N of Items
.858	54
